# Prognostic Factors for Residual Lithiasis in Patients With Staghorn Calculi Undergoing Percutaneous Nephrolithotomy in the Maya Region of Yucatan, Mexico: A Case-Control Study

**DOI:** 10.7759/cureus.57052

**Published:** 2024-03-27

**Authors:** Luis Roberto Garcia-Chairez, Carlos David Franco-Gonzalez, Carolina Aracelly Gonzalez-Guillermo, Mariana Mendez-Atoche, Carlos Miguel Sosa-Olivares, Eduardo Cruz Nuricombo, Jose I Robles Torres, Juan Pablo Flores-Tapia

**Affiliations:** 1 Urology, High Specialty Regional Hospital of the Yucatan Peninsula, Merida, MEX; 2 Clinical Sciences, Universidad Marista de Mérida, Merida, MEX; 3 Urology, Hospital Universitario Dr. José Eleuterio González, Monterrey, MEX

**Keywords:** percutaneous nephrolithotomy, lithiasis, staghorn calculi, urology surgery, stone-free rate, kidney stones, urolithiasis

## Abstract

Background: Yucatan stands out as the state with the highest prevalence of urolithiasis in Mexico, placing significant demands on healthcare services, such as consultation and surgical intervention. Staghorn calculi are related to recurrent urinary tract infections, and their management is always surgical. The stone-free rate is a parameter used to measure the success of surgery, with residual stones considered those persisting four weeks after surgical management. There are understudied prognostic factors that can predict the success of achieving stone-free status, taking into account the number of stones, their location, and the anatomical variations of the patient's collecting system. The study aims to determine the prognostic factors for residual lithiasis in patients with staghorn calculi treated with percutaneous nephrolithotomy at the High Specialty Regional Hospital of the Yucatan Peninsula.

Methods: A case-control study was performed including 188 patients, aged 18 years or older, and diagnosed with staghorn calculus from January 2022 to June 2023, grouping the patients according to their stone-free rate evidence on postoperative computed tomography. Data were collected from the records of the Urology Department at a high-specialty hospital in Yucatan. The groups were analyzed, aiming to establish an association between preoperative factors and postoperative outcomes measured in terms of stone-free rate.

Results: A total of 188 patients with staghorn calculi were included, with a predominance in females (58.5%) and a mean age of 45.4 ± 11.9 years. The most common comorbidity was hypertension (29.8%), and 27.7% had a history of recurrent urinary tract infections. Regarding the Sampaio classification, B1 was the most prevalent in our population with 66 cases (35.1%), while Type A2 was the least common (13.8%). According to what was obtained through the multivariate logistic regression model, the calyceal anatomy Type A1 and A2 were associated with residual lithiasis (*p*= 0.016 OR: 2.994 CI: 1.223-7.331), and Grade IV was associated with a higher rate of residual lithiasis (*p*=0.005 CI: 1.586-13.100). A statistically significant association was found between stone burden and the presence of residual lithiasis (*p*=< 0.001).

Conclusion: Guy’s Score Grade IV showed a higher incidence of residual lithiasis, seemingly associated with stone burden, leading to the conclusion that both factors were categorized as predictors for the development of post-surgical residual lithiasis. Regarding anatomical variations according to Sampaio, it was observed that types A1 and A2 showed a lower rate of stone-free status. Therefore, we also consider them as variables that may influence the achievement of success in endourological management. Personalized patient assessment allows for more accurate prognostic factors, enabling a more comprehensive surgical planning in the presence of staghorn calculi.

## Introduction

Renal lithiasis (RL) is a pathology that occurs when stones form due to changes in the solubility of urinary components, leading to oversaturation and crystal formation [1]. A Staghorn calculus is a large intrarenal calculus that fills the space of the renal collecting system, taking the shape of the renal calyces and pelvis, being potentially dangerous due to the difficulty it represents in surgical management [2]. These types of calculi can be classified according to their extent and obstruction as partial if they only affect part of the calyces, or complete when they fill the entire calyceal and collecting space [2]. 

The formation of staghorn calculi might be related to recurrent or chronic urinary tract infections caused by urease-producing bacteria, resulting in a concentration of magnesium ammonium phosphate, also known as struvite [3,4].

RL is a global condition that affects approximately 1-20% of the population, with a significant impact on individuals of working age, translating into high social and economic burdens [5]. In Mexico, Yucatan stands out with rates exceeding double the national average, with reports indicating that 5.8 out of every 10,000 inhabitants are affected, making it the first place in prevalence [6]. This increased prevalence is attributed to the significant incidence of metabolic abnormalities in the Maya population, with hypocitraturia being the predominant condition in both adults (61.9%) and children (35.8%) [7]. Specifically, staghorn calculi entail significant morbidity and mortality worldwide, as it can result in mortality rates of up to 28% in untreated cases [8]. 

Various techniques exist for managing RL, and the choice of the most suitable one is based on specific stone characteristics such as density, location, size, and number [9]. The treatment of staghorn calculi is always surgical and currently relies on minimally invasive techniques with the aim of achieving complete stone clearance and minimal morbidity [10]. Residual lithiasis is a factor that can be used to assess the success rate of endourological management, which is evaluated through tomography four weeks after undergoing surgery, as during this period natural spontaneous elimination occurs [11]. If after this period fragments ≤ 4 mm still exist, there is a high possibility of developing recurrent lithiasis and having to undergo a new intervention [12].

There are multiple classifications that allow us to predict the success of surgery, including predicting the risk of residual lithiasis based on the anatomy and characteristics of the calculi. Among the most commonly used scores is the Guy’s Stone Score (GSS), which allows us to know the complexity of lithiasis based on the number of calculi, their anatomical location, and the anatomy of the collecting system and also serves as an instrument to determine the most appropriate management for the patient and predict the success rate of surgery [13].

In the same way, it has been demonstrated that anatomical variations of the pyelocaliceal system have a significant impact on surgical management and outcomes, requiring the use of combined techniques to achieve a stone-free state in abnormal variations [14]. In order to have a more detailed and adequate classification of the collecting system, Sampaio and Mandarim-De-Lacerda proposed a system that classifies renal calyces that is currently the most accepted worldwide [15]. In our region, there are no descriptions of the GSS and Sampaio classification in relation to the stone-free rate, and furthermore, the characteristics of staghorn calculi in the Mayan population have not been accurately described. Therefore, the study aims to determine the prognostic factors for residual lithiasis in patients with staghorn calculi treated with percutaneous nephrolithotomy (PCNL) at the High Specialty Regional Hospital of the Yucatan Peninsula.

## Materials and methods

Study design and study population

A case-control study was conducted, characterized as an observational, retrospective, cross-sectional, and analytical investigation. The data were collected from the records of the Urology Department at the High Specialty Regional Hospital of the Yucatan Peninsula. The hospital's urology department handles a high demand of patients diagnosed with urolithiasis, coming from all states located in the southern region of Mexico. The study was conducted from January 2022 to June 2023. Aiming to establish an association between preoperative factors and postoperative outcomes measured in terms of stone-free rate, the following groups were analyzed: (i) the control group represented by patients with staghorn calculi who underwent PCNL without residual calculi evident on postoperative computed tomography (CT) scan; (ii) the case group represented by patients with staghorn calculi who underwent PCNL and had residual calculi post-procedure.

The postoperative outcome was measured in stone-free rate by abdominal plain CT scan up to one-month post-surgery, considering stones measuring equal to or less than 4 mm in their maximum diameter on CT scan as stone-free, while any stone larger than this measurement is considered residual calculi.

Inclusion and exclusion criteria 

We included 188 patients, comprising 110 females and 78 males, all aged 18 years or older and diagnosed with RL. These patients underwent surgery at the referral hospital between 2022 and 2023. Individuals lacking sufficient information in the database or urography records during their hospitalization were excluded from the analysis.

Data collection 

To collect sociodemographic characteristics, records and clinical notes from the hospital were reviewed from January 2022 to June 2023 extracting pertinent data for the study. 

Variables in the study

For the study's objectives, we aimed to outline the socio-demographic profile of the patients by incorporating categorical variables, including gender, obesity (BMI>30), and comorbidities such as hypertension, diabetes mellitus, chronic kidney disease, and recurrent urinary tract infections. The quantitative variables included age and body mass index (BMI).

**Figure 1:**
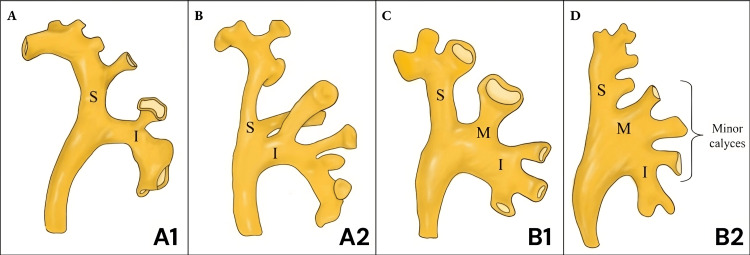
Sampaio classification. A) A1. Middle zone drains through minor calyces into superior (S) or inferior calyceal (I) group. B) A2. Middle zone drains through crossed calyces into superior (S) and inferior calyx (I). C) B1. Middle zone (M) drains into major calyx independently. D) B2. Middle zone (M) drains directly to the pelvis through minor calyces. Image credits: Mariana Mendez-Atoche, Carolina A. Gonzalez-Guillermo, Carlos D. Franco-Gonzalez

Regarding the stone characteristics, the following categorical variables were included: complete stone calculi, stone laterality, GSS grades, and hydronephrosis, and patients were classified based on preoperative pyelography according to the Sampaio's Classification into types A1, A2, B1, and B2 (Figure 1) [15]. Additionally, the following quantitative variables were included: Stone Burden (mm3), GSS, stone-to-skin distance, and stone density (Hounsfield Units).

Statistical analysis

Descriptive and inferential statistics were performed using IBM SPSS Statistics for Windows, Version 24 (Released 2016; IBM Corp., Armonk, New York, United States). Categorical variables were represented in frequencies and percentages, while numerical variables were represented in mean and standard deviation. Normality tests were conducted to determine the statistical test for numerical variables. For categorical variables, the Chi-squared test was utilized to determine factors associated with residual lithiasis. Additionally, the Mann-Whitney U test was employed for numerical variables with non-normal distribution. Univariate analysis was conducted, and variables with statistical significance were included in a multivariate regression model.

Ethical considerations

Ethical approval was obtained from the Ethics Committee of the High Specialty Regional Hospital of the Yucatan Peninsula with code 2023-018 on October 20, 2023.

## Results

The sociodemographic data and comorbidities of the studied population are illustrated in Table 1. A total of 188 patients with staghorn calculi were obtained, with females comprising a clear majority (n=110; 58.5%) compared to males (n=78; 41.5%). The mean age at the time of surgery was 45.4 years ± 11.9 years, ranging from 19 to 82 years. Of the total, the most frequently encountered comorbidities were hypertension (29.8%), followed by Type 2 diabetes mellitus and chronic kidney disease at 19.7% and 17.6%, respectively. Staghorn calculi were more prevalent in obese patients (BMI ≥30), accounting for 41% (n=77). A notable feature in our study population is that 27.7% of patients had a history of recurrent urinary tract infections (Table 1).

**Table 1:** Sociodemographic, clinical, metabolic, and microbiological characteristics of the study population (n=188).

Variable	Number	Percentage	Mean ± SD
Age (years)			45.4 ± 11.9
Male	78	41.5%	
Female	110	58.5%	
Body mass index			28.4 ± 15.5
Obesity (BMI>30)	77	41%	
Comorbidities			
Hypertension	56	29.8%	
Diabetes mellitus	37	19.7%	
Chronic kidney disease	33	17.6%	
Recurrent urinary tract infections	52	27.7%	

The majority of stones were found in the right kidney, with 112 (59.6%) compared to the left kidney with 76 (40.4%). According to the Sampaio classification, the predominant pelvicalyceal pattern was Type B1, comprising a total of 66 cases (35.1%). Subsequently, Type A1 accounted for 53 cases (28.2%), Type B2 for 43 cases (22.9%), and Type A2 presented the lowest number of cases, accounting for only 26 (13.8%). Details related to these classifications are summarized in Table 2.

**Table 2:** Classification according to Sampaio and Stone characteristics of the study population (n=188). *Type A1: Middle zone drains through minor calyces into superior or inferior calyceal group *Type A2: Middle zone drains through crossed calyces into superior and inferior calyx *Type B1: Middle zone drains into major calyx independently *Type B2: Middle zone drains directly to the pelvis through minor calyces

Variable	Number	Percentage	Mean ± SD
Sampaio´s Classification			
Type A1	53	28.2%	
Type A2	26	13.8%	
Type B1	66	35.1%	
Type B2	43	22.9%	
Stone characteristics			
Complete Staghorn Calculi	87	46.3%	
Calculi in left kidney	76	40.4%	
Calculi in right kidney	112	59.6%	
Stone Burden (mm^3^)			1061.5 ± 763.2
Guy's Stone Score			2.86 ± 1.01
Guy's Stone Score I	24	12.8%	
Guy's Stone Score II	39	20.7%	
Guy's Stone Score III	65	34.6%	
Guy's Stone Score IV	60	31.9%	
Hydronephrosis	75	39.9%	
Stone-to-skin distance			12 ± 2.01
Stone Density (Hounsfield Units)			1059.9 ± 377.4

The average stone volume was 1061.5 ± 763.2 mm², the stone-to-skin distance had a mean of 12 cm, stone hardness measured by Hounsfield Units on tomography averaged 1059.9, and hydronephrosis was present in 39.9% of the studied patients. 

In the multivariate logistic regression model, it was determined that stones classified as GUY 4 were associated with a higher rate of residual lithiasis with statistically significant difference (p= 0.005, CI [1.586-13.100]). No significant differences were found in lower grades on the GUY scale. On the other hand, categorizing patients based on Sampaio's classification, a difference was found in the stone-free rate depending on the anatomy of the renal calices. Calyceal anatomy Type A1, where minor calyces in the midzone drain dependent on a major calyx, and calyceal anatomy Type A2, where the midzone drains simultaneously via crossing calices were factors associated with residual lithiasis with a statistically significant difference (p= 0.016, OR 2.994 CI [1.223-7.331]), (p= 0.002, OR 13.769 CI [2.712-69.916]) (Table 3).

**Table 3:** Multivariable logistic regression of factors associated with stone-free rate (n=188). ^*^The significance level is p<0.05 (significant) using the Chi- square test for the univariate analysis ^**^The significance level is p<0.05 (significant) using the Chi- square test for the multivariate analysis

			Univariate		Multivariate	
Variable	Stone-free (n=94)	Not Stone-free (n=94)	p-value	OR (IC 95%)	p-value	OR (IC 95%)
Sampaio´s Classification						
Type A1	19 (20.2)	34 (36.2)	0.023*	2.237 (1.161-4.311)	0.016**	2.994 (1.223-7.331)
Type A2	2 (2.1)	24 (25.5)	<0.001*	15.771 (3.606-68.980	0.002**	13.769 (2.712-69.916)
Type B1	46 (48.9)	20 (21.3)	<0.001*	0.282 (0.149-0.534)	0.577	.779 (0.324-1.874)
Type B2	27 (28.7)	16 (17)	0.082	0.509 (0.253-1.024)		
Stone characteristics						
Complete Staghorn Calculi	37 (39.4)	50 (53.2)	0.079	0.571 (0.320-1.019)		
Guy's Stone Score I	15 (15.9)	9 (9.5)	Reference	Reference		
Guy's Stone Score II	39 41.5)	26 (27.7)	0.781	1.159 (0.408-3.294)	0.806	1.149 (0.378-3.488)
Guy's Stone Score III	23 (24.4)	16 (17)	0.83	1.111 (0.424-2.913)	0.648	1.268 (0.457-3.524)
Guy's Stone Score IV	17 (18)	43 (45.7)	0.005*	4.216 (1.552-11.449)	0.005**	4.558 (1.586-13.100)
Hydronephrosis	33 (35.1)	42 (44.7)	0.233	0.670 (0.372-1.205)		

Continuous variables were analyzed separately as shown in Table 4, finding that certain stone characteristics on tomography such as stone burden (mm^3^) were associated with post-procedure residual lithiasis, being statistically significant (p=<0.001). There was no statistically significant difference in surgical outcome regarding the stone-to-skin distance or stone hardness measured by Hounsfield Units on tomography (Table 4).

**Table 4:** . Evaluation of numerical variables for the stone-free rate. *The significance level is p<0.05 (significant) using the Chi-square test

Variables	Stone-free (n=94)	Not Stone-free (n=94)	p-value
Stone characteristics			
Stone burden (mm^3^)	883.23 ± 635	1239 ± 838	<0.001*
Stone-to-skin distance	11.9 ± 2.11	12.1 ± 1.9	0.68
Stone density (Hounsfield Units)	1075.9 ± 427.3	1043.8 ± 321.3	0.561

## Discussion

Urinary lithiasis constitutes a significant public health issue with a considerable impact on working-age patients, particularly in the state of Yucatan, Mexico. A special case is the staghorn calculi, whose treatment is always surgical; however, achieving a stone-free state can be challenging and may require staged or combined approaches, leading to increased hospitalizations, a rise in surgical events, and a risk of complications related to patient medical and surgical care, as well as a significant increase in economic expenditure for both the population and healthcare services in our country.

Among the variables in our population, there was similarity regarding the average age of presentation of this pathology compared to the literature, being more common in the fifth decade of life [16]. There are aspects that differ from those reported, such as the predisposition in terms of gender, being higher in females in our population (58.5%), contrary to the demographic characteristics, where up to twice the risk is reported in males compared to females [17,18].

Nonetheless, there are similar characteristics such as some comorbidities that, due to their pathophysiology predispose to a greater risk of lithiasis, such as hypertension since these patients may present greater excretion of urinary calcium than normal tense patients [19]. Hypertension is the second comorbidity most associated with the formation of staghorn stones in 29.8% of patients. Additionally, obesity emerged as the comorbidity predominantly related to this pathology, with a frequency similar to that reported by Sansores-España around 47%, being found in 41% of cases in our study [16]. 

Our research examined the relationship between the complexity of renal stones, as evaluated by the GUY scoring system, and the outcomes of PCNL. This score, well-regarded for its comprehensive assessment of stone complexity based on factors such as the number of involved calyces and specific calyceal anatomical features, suggests that higher grades correlate with increased surgical complexity and the likelihood of residual stones. Our study confirmed this association solely for GSS IV, indicative of complete staghorn calculi, which carry a heightened risk of residual stones. Our findings align with those of de Souza Melo et al., who, after analyzing 1,066 PCNL procedures in 891 patients, observed a success rate decreasing inversely with calculus complexity, particularly noting a 19.5% success rate in GSS4 cases [20]. Similarly, Rashid et al. reported consistent results in 115 patients undergoing PCNL, wherein CT-based GSS accurately predicted success rates and postoperative complications [21].

Considering the aforementioned, standardizing the presentation of calyceal anatomy is essential, as according to the findings of our study, there are significant differences in the various variants of the renal calyceal system according to the Sampaio classification. Our study more frequently observed Type B1, similar to what Marroig et al. reported, where this subgroup was obtained in 34.71% of 170 cadaveric kidneys of Brazilian origin [22]. In contrast, other studies found that the most commonly encountered morphology was Type A1, with an occurrence ranging from 30.5% to 64% [15,23-26]. These variations may be attributed to the local characteristics of the diverse ethnic composition in our study population, emphasizing the need for additional research in the region to explore potential genetic factors associated with these observations.

Regarding the stone-free rate, it was found that patients with a pyelocaliceal anatomy according to Sampaio A1 and A2 were prognostic factors for higher postoperative residual lithiasis. Similar results were reported in the study by Kirecci et al., in their analysis of 125 flexible ureteroscopies, they evaluated the stone elimination rate in all subgroups of cases according to the Sampaio classification, where it was found that the rate of Stone clearance was significantly lower in subgroup A2 at 30.4%, while it was significantly higher in subgroup B2 reaching 93.8% [14].

The stone burden obtained falls within the reported ranges in the literature and stands out as a factor impacting residual lithiasis, which is consistent with findings where residual burden is the most important factor for residual lithiasis presence [22,27]. Similarly, these findings appear to be linked to a higher incidence of residual lithiasis observed in patients with complete staghorn calculi (GSS 4).

The primary limitations encountered in this study were attributed to the absence of prior research on lithiasis and anatomical variations in the region and country.

## Conclusions

According to the GUY score classification, we observed a prognostic factor of a higher incidence of residual lithiasis in patients with complete coral (GSS 4), seemingly correlated with lithiasic burden, which was also found as a factor for postoperative residual lithiasis. However, lower GUY scores (GSS 2 and 3), which account for anatomical anomalies, were not found to be associated with residual lithiasis.

The analysis of renal calyceal variants according to the Sampaio classification revealed Type B1 as the most frequent variant in our sample, followed by A1. Additionally, Types A1 and A2 were associated with a lower stone-free rate.

This study provides important insights into predictive factors that can improve personalized patient care and surgical planning for staghorn calculi. By optimizing resource allocation, the goal is to maximize patient benefit and utilize technological advancements for diagnosing and treating coraliform lithiasis. The implications of this analysis go beyond enhancing medical care; it also opens doors for further research in the field, ultimately advancing our understanding and management of this condition.

## References

[1] Khan A (2018). Prevalence, pathophysiological mechanisms and factors affecting urolithiasis. Int Urol Nephrol.

[2] Terry RS, Preminger GM (2020). Metabolic evaluation and medical management of staghorn calculi. Asian J Urol.

[3] Preminger GM, Assimos DG, Lingeman JE, Nakada SY, Pearle MS, Wolf JS Jr (2005). Chapter 1: AUA guideline on management of staghorn calculi: diagnosis and treatment recommendations. J Urol.

[4] Griffith DP, Gibson JR, Clinton CW, Musher DM (1978). Acetohydroxamic acid: clinical studies of a urease inhibitor in patients with Staghorn renal calculi. J Urol.

[5] Alexander RT, Fuster DG, Dimke H (2022). Mechanisms underlying calcium nephrolithiasis. Annu Rev Physiol.

[6] Medina-Escobedo M, Zaidi M, Real-de León E, Orozco-Rivadeneyra S (2002). Prevalencia y factores de riesgo en Yucatán, México, para litiasis urinaria. Salud Publica Mex.

[7] Medina-Escobedo M, Franco-Bocanegra D, Villanueva-Jorge S, González-Herrera L (2013). The I550V polymorphism in the renal human sodium/dicarboxylate cotransporter 1 (hNaDC-1) gene is associated with the risk for urolithiasis in adults from Southeastern, Mexico. Open J Genet.

[8] Koga S, Arakaki Y, Matsuoka M, Ohyama C (1991). Staghorn calculi--long-term results of management. Br J Urol.

[9] Fisang C, Anding R, Müller SC, Latz S, Laube N (2015). Urolithiasis--an interdisciplinary diagnostic, therapeutic and secondary preventive challenge. Dtsch Arztebl Int.

[10] Seitz C, Desai M, Häcker A, Hakenberg OW, Liatsikos E, Nagele U, Tolley D (2012). Incidence, prevention, and management of complications following percutaneous nephrolitholapaxy. Eur Urol.

[11] Almeras C, Raynal G, Meria P (2023). 2022 recommendations of the AFU Lithiasis Committee: objectives, results, residual stones and fragments. Prog Urol.

[12] Brain E, Geraghty RM, Lovegrove CE, Yang B, Somani BK (2021). Natural history of post-treatment kidney stone fragments: a systematic review and meta-analysis. J Urol.

[13] Thomas K, Smith NC, Hegarty N, Glass JM (2011). The Guy’s Stone score—grading the complexity of percutaneous nephrolithotomy procedures. Urol.

[14] Kirecci SL, Ilgi M, Yesildal C, Yavuzsan AH, Albayrak AT, Sarica K (2021). The impact of the pelvicalyceal anatomy characteristics on the prediction of flexible ureteroscopy outcomes. Urol Ann.

[15] Sampaio FJ, Mandarim-De-Lacerda CA (1988). Anatomic classification of the kidney collecting system for endourologic procedures. J Endourol.

[16] Sansores-España DJ, Medina-Escobedo MMÁ, Rubio-Zapata HA, Romero-Campos SG, Leal-Ortega G (2020). Metabolic syndrome and urolithiasis: a case-control study (Article in Spanish). Rev Med Inst Mex Seguro Soc.

[17] Aune D, Mahamat-Saleh Y, Norat T, Riboli E (2018). Body fatness, diabetes, physical activity and risk of kidney stones: a systematic review and meta-analysis of cohort studies. Eur J Epidemiol.

[18] Ziemba JB, Matlaga BR (2017). Epidemiology and economics of nephrolithiasis. Investig Clin Urol.

[19] Shang W, Li Y, Ren Y, Yang Y, Li H, Dong J (2017). Nephrolithiasis and risk of hypertension: a meta-analysis of observational studies. BMC Nephrol.

[20] de Souza Melo PA, Vicentini FC, Beraldi AA, Hisano M, Murta CB, de Almeida Claro JF (2018). Outcomes of more than 1 000 percutaneous nephrolithotomies and validation of Guy's stone score. BJU Int.

[21] Rashid AO, Khalid H, Friad G, Hamed RY, Buchholz N (2020). Guy's Stone score as a predictor for stone-free rate and complications in percutaneous nephrolithotomy: a single-center report from a Stone belt country. Urol Int.

[22] Marroig B, Favorito LA, Fortes MA, Sampaio FJ (2015). Lower pole anatomy and mid-renal-zone classification applied to flexible ureteroscopy: experimental study using human three-dimensional endocasts. Surg Radiol Anat.

[23] Anjana TS, Muthian E, Thiagarajan S, Shanmugam S (2017). Gross morphological study of the renal pelvicalyceal patterns in human cadaveric kidneys. Indian J Urol.

[24] Çiçek R, Dündar G, Gökçen K, Gökçe G, Gültekin EY (2022). The evaluation of morphology of renal pelvicalyceal system's and infundibulopelvic anatomy of kidney's lower pole in post-mortem series. Folia Morphol (Warsz).

[25] Talyshinskii A, Guliev B, Komyakov B, Galfano A (2020). Patient counseling through the pelvicalyceal-shaped labyrinth: in search of an easy understanding of the upcoming stone removal: a pilot study. Urology.

[26] Yazici O, Binbay M, Akman T (2013). Is there a difference in percutaneous nephrolithotomy outcomes among various types of pelvicaliceal system?. World J Urol.

[27] Okhunov Z, Friedlander JI, George AK (2013). S.T.O.N.E. nephrolithometry: novel surgical classification system for kidney calculi. Urology.

